# Comparison of theoretical fixation stability of three devices employed in medial opening wedge high tibial osteotomy: a finite element analysis

**DOI:** 10.1186/1471-2474-15-230

**Published:** 2014-07-10

**Authors:** Мaxim L Golovakhа, Weniamin Orljanski, Karl-Peter Benedetto, Sergey Panchenko, Philippe Büchler, Philipp Henle, Emin Aghayev

**Affiliations:** 1 Department of Orthopaedic Surgery, Zaporozhye State Medical University, Mayakovskyi avenue 26, Zaporozhye, Zaporozhye Oblast 69035, Ukraine; 2 Department of Orthopaedic Surgery, Vienna Private Clinic, Pelikangasse 15, Vienna 1090, Austria; 3 Department of Traumatology, Landeskrankenhaus Feldkirch, Carinagasse 47, Feldkirch 6807, Austria; 4 Institute for Surgical Technology and Biomechanics, University of Bern, Stauffacherstrasse 78, Bern 3014, Switzerland; 5 Prydniprovs’ka State Academy of Civil Engineering and Architecture, 1997-2013 24a, Chernyshevs’kogo St, Dnipropetrovs’k 49600, Ukraine; 6 Department of Orthopaedic Surgery, Sonnenhof Hospital, Buchserstrasse 30, Bern 3006, Switzerland; 7 Institute for Evaluative Research in Medicine, University of Bern, Stauffacherstrasse 78, Bern 3014, Switzerland

**Keywords:** High tibial osteotomy, Open wedge osteotomy, Puddu plate, TomoFix plate, Tibial osteotomy fixation, Comparative osteosynthesis stability

## Abstract

**Background:**

Medial open wedge high tibial osteotomy is a well-established procedure for the treatment of unicompartmental osteoarthritis and symptomatic varus malalignment. We hypothesized that different fixation devices generate different fixation stability profiles for the various wedge sizes in a finite element (FE) analysis.

**Methods:**

Four types of fixation were compared: 1) first and 2) second generation Puddu plates, and 3) TomoFix plate with and 4) without bone graft. Cortical and cancellous bone was modelled and five different opening wedge sizes were studied for each model. Outcome measures included: 1) stresses in bone, 2) relative displacement of the proximal and distal tibial fragments, 3) stresses in the plates, 4) stresses on the upper and lower screw surfaces in the screw channels.

**Results:**

The highest load for all fixation types occurred in the plate axis. For the vast majority of the wedge sizes and fixation types the shear stress (von Mises stress) was dominating in the bone independent of fixation type. The relative displacements of the tibial fragments were low (in μm range). With an increasing wedge size this displacement tended to increase for both Puddu plates and the TomoFix plate with bone graft. For the TomoFix plate without bone graft a rather opposite trend was observed.

For all fixation types the occurring stresses at the screw-bone contact areas pulled at the screws and exceeded the allowable threshold of 1.2 MPa for at least one screw surface. Of the six screw surfaces that were studied, the TomoFix plate with bone graft showed a stress excess of one out of twelve and without bone graft, five out of twelve. With the Puddu plates, an excess stress occurred in the majority of screw surfaces.

**Conclusions:**

The different fixation devices generate different fixation stability profiles for different opening wedge sizes. Based on the computational simulations, none of the studied osteosynthesis fixation types warranted an intransigent full weight bearing per se. The highest fixation stability was observed for the TomoFix plates and the lowest for the first generation Puddu plate. These findings were revealed in theoretical models and need to be validated in controlled clinical settings.

## Background

Medial opening wedge high tibial osteotomy (MOWHTO) is a well-established procedure for the treatment of unicompartmental osteoarthritis and symptomatic varus malaligned knees [1,2]. Early weight bearing after corrective tibial osteotomy has been regarded as desirable [2]. In the last decade there has been a renaissance of MOWHTO catalysed by the use of angle stabile plates with locking head screws (LHS) [3-7].

Several fixation types for the MOWHTO are currently used and several research groups worked on the assessment of their fixation stability in biomechanical experiments [7-12]. The two most frequently used implants are the Puddu and the TomoFix systems [12]. The Puddu plate uses the dynamic compression concept, whereas the TomoFix plate follows the locking compression concept. Raja Izaham et al. have recently compared fixation stability of these two systems using FE analysis [13]. According to the authors, the model of the TomoFix plate fixation resulted in a higher fixation stability; however, the bone model in the study was based upon cortical bone properties only for saving calculation time [13]. Another FE analysis by Luo et al. compared TomoFix plate, T plate, T + I plate, and π plate regarding the occurring stresses and stability in MOWHTO [14]. The authors concluded that TomoFix and T plate as the so-called one-leg systems with LHS can be used for the majority of patients without heavy bodyweight and poor bone quality. Both FE analyses simulated only one opening wedge size [13,14].

The use of bone graft to increase mechanical strength remains controversial; whilst it may increase mechanical strength, some authors have suggested that opening wedges can be left unfilled [3], which avoids the risk associated with gap filling with inert substances [15]. Zorzi et al. tried to assess the effect of bone graft versus no graft [16]. There was no significant difference in time to bone healing between the patient groups, though correction loss was twice as common in the group without bone graft (n = 2 versus n = 1). But the low occurrence rate of this event did not result in a significant difference between the groups. According to the authors larger studies are required for the assessment of correction loss occurrence rates in patients with and without bone graft filling [16].

FE analysis has gained wide acceptance in orthopaedic research as a technique which simulates bone-implant constructs and physiological loads aimed at predicting the outcome of surgery [13,14]. FE analysis is a numerical method to solve differential equations by discretization of the shape of complicated domains into a finite number of well-defined elements. The contribution of each element is assembled to form a system of equations that can be solved numerically using iterative techniques [17].

The current study aimed at FE-based quantitative comparison of the fixation stability between the most frequently used plates with different fixation characteristics. The effect of gap filling was studied for one implant. We hypothesized that the different fixation devices generate different fixation stability profiles for various opening wedge sizes in a finite element analysis.

## Methods

**Figure 1:**
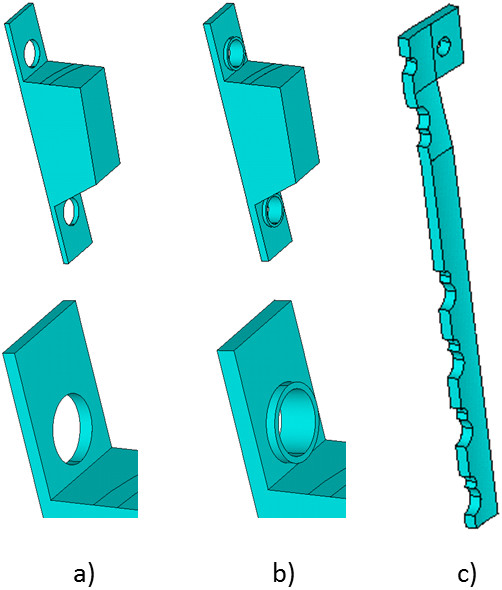
**Model of plates. a)** First generation Puddu plate, **b)** Second generation Puddu plate, **c)** TomoFix plate with locking head screws.

No ethical committee approval or patient consent was required for this theoretical study on simulated bone-implant constructs and physiological loads.The study was performed based on the FE analysis method using ANSYS software (Version 14.5, Ansys Inc.). For the purpose of rational use of computer resources a geometric plane of symmetry and strength of the “bone-plate” calculation models was assumed. Four models of fixation were analysed: 1) first generation Puddu plate, 2) second generation Puddu plate with LHS, 3) first generation TomoFix plate with LHS without bone graft, and 4) first generation TomoFix plate with LHS and bone graft (Figure 1a-c).

The first generation Puddu plate is a stainless steel plate with an incorporated metallic block for propping the distracted medial corticalis [12]. The second generation Puddu plate design differs from the first due to having LHS holes, through which the screws can be freely orientated in any direction and then locked into the plate [18]. The first generation TomoFix plate is a t-shape plate with three horizontally arranged locking screw holes in the proximal part and five combination holes in the longitudinal part of the plate [3]. The plate is intended to hold the attained correction without additional bone substitutes filling the osteotomy gap [3].

### Modelling of tibia and implants

The plate models were built using AutoCAD (Version 2012, Autodesk) software by constructing separate cross-sections of bone and plates. The geometry of the bone cross-sections was based upon a computerized tomography (CT) scan of a 40-year-old healthy man of 180 cm height and 85 kg weight with an asymptomatic knee joint. The tibia bone model ended 140 mm below the joint line. At the following levels, counting from distal to proximal, bone cross-sections in the frontal and sagittal planes were determined: h1 = 0 mm, h2 = 65 mm, h3 = 105 mm, h4 = 120 mm, h5 = 140 mm.

During modelling the real shape of the cross-sections was replaced by ellipses whose axes were obtained from the above mentioned CT scan. The bone tissue was modelled as a continuous piecewise homogeneous isotropic medium. The proportions of cortical layer and cancellous bone were reproduced in the according cross-sections based on the measurements from the CT scan. The thickness of the cortical layer varied from a maximum of 5.0 mm in the lowest cross-section h0 to 1.0 mm in the cross-section h4 at 120 mm. The cortical layers between the h0 and h4 levels were endowed with the modulus of elasticity E = 2 × 10^4^ MPa and Poisson's ratio ν = 0.3 that were constant through the whole length [13,19]. For the uppermost 20 mm between the h4 and h5 levels, the cortical layer was not modelled because of its thinness and its resulting properties being similar to cancellous bone. The cancellous bone was divided into three parts with the averaged elastic properties of the real tibia between h0 and h1 - E = 200 MPa and ν = 0.3, between h1 and h4 - E = 500 MPa and ν = 0.3, and between h4 and h5 - E = 800 MPa and ν = 0.3 [20].Plates and screws were modelled according to their real sizes. Screw holes in the plates were identical. Each hole was supplied with a chamfer for the head of the screw. The shape of the plates was replaced by a symmetric half. The first generation Puddu plate had the dimensions 17×47×1 mm. The Puddu plate with LHS differed from the first generation plate by 1.5 mm thickness and screw holes, which had cylindrical inserts (Figure 1b). These screw holes were modelled as cylinders with an outer diameter of 6 mm, an inner diameter of 5 mm, and a height of 0.75 mm on each side of the plate (Figure 1b). The TomoFix plate had a length of 114 mm; the width of the top was 34 mm and of the bottom 16 mm; the thickness was 2.5 mm. The metal inset for the Puddu plates was modelled in the form of a truncated wedge with the same width as the plate (17 mm), depth of insertion (starting from the inner surface of the plate) - 10 mm, whereas this depth was varying between 3 mm on the outer side of the insert and 8 mm in the plane of symmetry. The insert for the bone graft for the TomoFix model was also modelled as a truncated wedge with a width of 20 mm and a depth between 14 mm on the outer side and up to 20 mm in the plane of the symmetry. Since the insertion was modelled in the form of a truncated wedge, its height was variable and corresponding to the size of the wedge gap.

The lengths of the screws ranged between 20 and 50 mm, the diameter of the screws was 5.0 mm for the Puddu plates and 4.0 mm for the TomoFix plates.

The plates were mounted on the bone model with a 2 mm gap to account for soft tissues like periosteum. The characteristics of the plates and screw material were E = 2 × 10^5^ MPa and ν = 0.3. Since the characteristics of bone grafts may vary between cancellous and cortico-cancellous bone properties and also between different patients, the following averaged parameters were selected E = 800 MPa and ν = 0.3.

The connection between the LHS screws and the second generation Puddu and the TomoFix plates was of a hard type. In the model of the first generation Puddu plate, the screws and the plate were connected by imperfect joints, which were modelled by a reduction in the local bending stiffness of the screw in its connection to the plate. This stiffness reduction was achieved by inclusion of an additional insert consisting of a truncated cone and the cylinder adjacent to the lower base of the cone. The length of the cone was 1.0-1.5 mm, and the diameter and length of the cylinder were 0.9 mm and 0.15-0.35 mm, respectively. That way the yielding capacity of the screw with respect to the transverse load was considerably higher. Since the real magnitude of the stiffness reduction of the screw fixation in the first generation Puddu plate is, in principle, unclear, the model of imperfect joint was regarded as sufficient. The screw-bone fixation was simulated with a full grip between the smooth surface of the screw and the smooth surface of the bone holes in the area of contact.

The bone osteotomy was modelled by a sphenoid gap. The upper plane of the osteotomy wedge had an angle of 15° to the horizontal plane. Five following opening wedge sizes for each of the models were studied: 7.5, 10.5, 12.5, 17.5, and 22.5 mm. The computational models were fixed on the entire plane of the lower base, whereas restrictions were imposed for vertical and horizontal displacements. The corresponding displacement restrictions were imposed in the plane of symmetry of the model.

**Figure 2:**
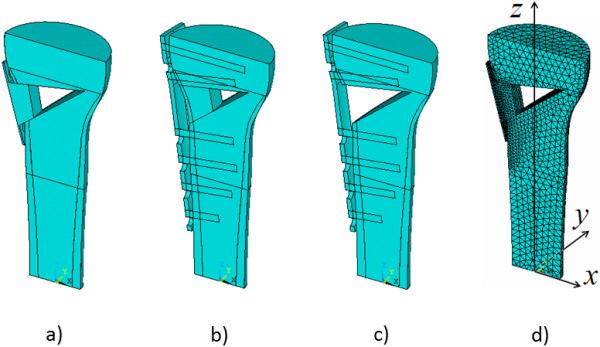
Calculation of bone-plate models (a, b, c) and FE model (d).

The loading was carried out by the application of a pressure of 0.3 MPa to the entire area of the upper base of the model. This corresponds to a resultant force of 800 N and resembles full weight bearing.The FE models were calculated on the basis of a 10-node FE in the form of a tetrahedron Solid186, with three degrees of freedom at each node. Dimensions of the FE were given along the lines ranging from 8 mm at the far boundaries to 1 mm in screws and 0.8 mm near the edge of the holes. To account for structural non-linearity that occurs in the screw-bone contact area, a contact pair was created for all screws using the menu of Contact Manager. 3D contact surfaces were generated using Targe 170 and Conta 175 for screws and bones, respectively. In total, between 30’000 and 40’000 FE were used depending on the model for stable calculation of the results. For the more accurate results, the function Substep was used for a consistent increase in applied load during computations. The calculated FE models and the used coordinate system are shown in Figure 2.

### Outcome measures

Outcome measures included: 1) stresses in bone, 2) relative displacement of the proximal and distal tibial fragments, 3) stresses in the plates, and 4) stresses on the upper and lower screw surfaces in the screw channels along the X-axis (Figure 2).

1.2 MPa was used as the maximum allowable threshold for the stresses on the screw. The value was derived from the 842 N as the screw extraction strength resulting in a screw fixation failure [21] applied to a 50 mm long screw (the maximum screw length modelled) with a diameter of 4.5 mm (the average of 4 and 5 mm modelled screws).

## Results

### Stresses in bone

**Figure 3:**
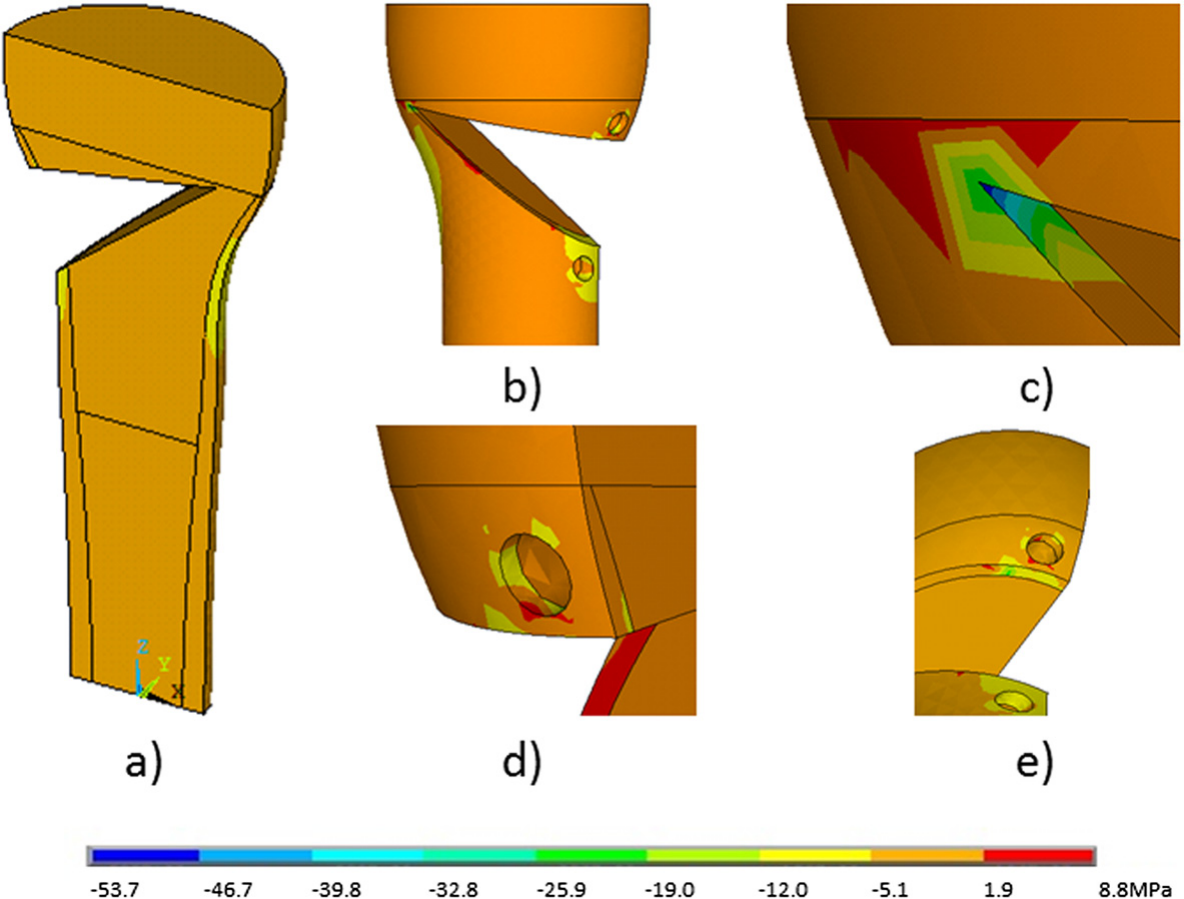
**The distribution of stresses in the σ**_**z **_**plane in the bone in the entire volume (a) and in the zones of stress concentration (b-e) for the osteosynthesis with the first generation Puddu plate.**

The patterns of stresses in bone were close to uniform except for zones of stress concentration like sharp corners and edges (Figure 3). At these points a substantial deviation in the values of the stresses was observed. In general, the pattern of stress distributions in 3D in bone was similar for all opening wedge sizes and plates. For the vast majority of the opening wedge sizes and fixation types the shear stress (von Mises stress), which was acting perpendicular to the weight bearing axis Z, had the highest magnitudes (Table 1).

**Table 1:** **The stresses in bone at the corresponding points in brackets (s. Figure **4**) at different wedge openings**

**Fixation**	**Wedge opening (mm)**	_**max**_**σ**_**z **_**(tensile stress)**	_**min**_**σ**_**z **_**(compression stress)**	_**max**_**σ**_**x **_**(tensile stress)**	_**min**_**σ**_**x **_**(compression stress)**	_**max**_**σ**_**y **_**(tensile stress)**	_**min**_**σ**_**y **_**(compression stress)**	_**Mis**_**σ (shear von Mises stress)**
First generation Puddu plate	7.5	8.0 (a)	−23.7 (b)	15.9 (a)	−6.6 (a)	15.1 (c)	−35.9 (b)	45.8 (b)
	10.5	3.6 (a)	−27.2 (b)	11.4 (a)	−19.9 (b)	14.7 (c)	−40.7 (b)	39.6 (b)
	12.5	4.4 (c)	−28.0 (b)	11.6 (a)	−22.4 (b)	12.9 (c)	−40.0 (b)	40.5 (b)
	17.5	4.8 (c)	−27.1 (b)	11.9 (a)	−17.9 (b)	12.4 (c)	−38.5 (b)	50.9 (b)
	22.5	3.0 (c)	−26.9 (a)	11.5 (a)	−6.6 (a)	8.9 (c)	−26.1 (b)	29.2 (b)
Second generation Puddu plate	7.5	3.3 (a)	−25.9 (b)	10.8 (a)	−15.3 (b)	6.8 (c)	−34.4 (b)	23.6 (b)
	10.5	3.5 (a)	−27.3 (b)	10.9 (a)	−18.0 (b)	8.5 (c)	−32.2 (b)	38.4 (b)
	12.5	3.4 (a)	−29.1 (b)	10.9 (a)	−19.2 (b)	7.0 (c)	−33.9 (b)	39.5 (b)
	17.5	3.3 (a)	−29.9 (b)	11.4 (a)	−18.5 (b)	8.1 (c)	−31.2 (b)	40.5 (b)
	22.5	2.6 (c)	−26.1 (a)	10.9 (a)	−6.4 (a)	5.8 (c)	−25.4 (b)	29.9 (b)
TomoFix plate with bone graft	7.5	7.1 (c)	−23.6 (a)	12.4 (a)	−6.4 (d)	6.2 (c)	−10.6 (d)	25.4 (a)
	10.5	12.9 (c)	−29.9 (a)	8.88 (a)	−5.9 (d)	5.4 (c)	−10.4 (d)	32.0 (a)
	12.5	6.7 (c)	−29.9 (a)	9.16 (a)	−5.8 (d)	4.4 (c)	−10.4 (d)	32.7 (a)
	17.5	7.7 (c)	−20.5 (a)	9.68 (a)	−6.3 (d)	5.4 (c)	−11.1 (d)	23.0 (a)
	22.5	5.6 (d)	−23.8 (a)	11.0 (a)	−6.1 (d)	4.7 (c)	−11.6 (d)	27.2 (a)
TomoFix plate without bone graft	7.5	21.1 (a)	−25.8 (a)	13.2 (a)	−7.2 (d)	8.6 (c)	−12.9 (d)	25.8 (a)
	10.5	7.8 (c)	−33.2 (a)	13.8 (a)	−7.6 (d)	4.8 (c)	−13.4 (d)	31.3 (a)
	12.5	6.7 (c)	−32.7 (a)	13.9 (a)	−7.3 (d)	7.0 (c)	−13.2 (d)	31.5 (a)
	17.5	6.1 (c)	−31.4 (a)	11.4 (a)	−6.5 (d)	7.5 (c)	−14.1 (d)	31.7 (a)
	22.5	14.8 (a)	−24.1 (a)	13.4 (a)	−7.4 (c)	5.7 (c)	−14.9 (d)	25.7 (a)

**Figure 4:**
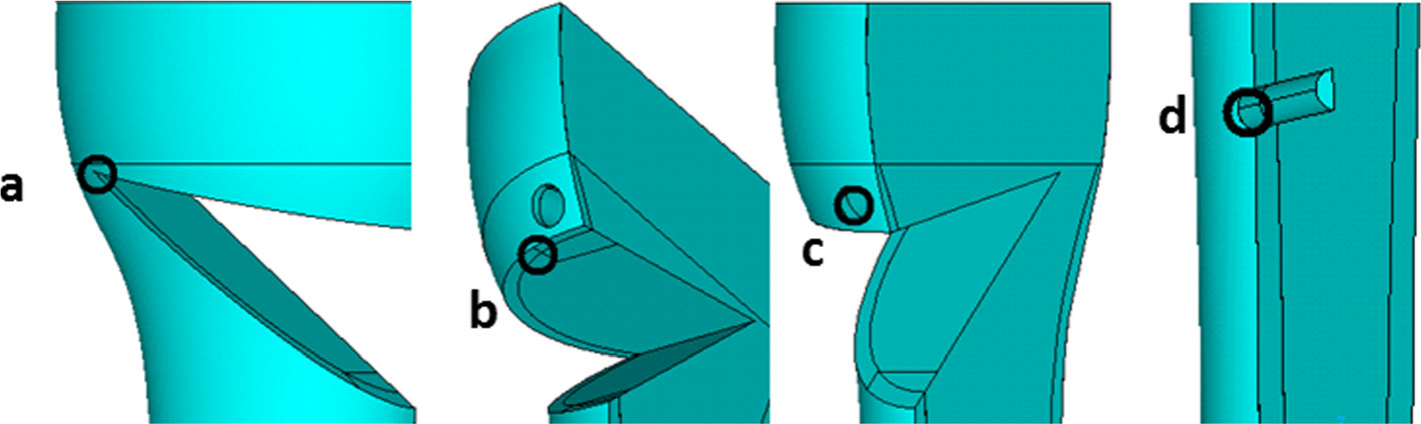
**Localization of the stresses for Puddu (a-c) and TomoFix plates (a, c, d) specified in Table **1**. a)** The point at the conjunction between the proximal and distal tibia fragment; **b)** The contact point of the proximal bone fragment and the metal insert; **c)** The point at the screw hole just above the osteotomy; **d)** The point at the edge of the most distal screw hole in the distal tibia fragment.

For both Puddu plates the compressive stresses in the Y-axis had the second highest magnitudes (Table 1) occurring in the bone-plate contact point between the upper tibial fragment and the Puddu plate insert (Figure 4). In the Puddu plate with LHS, the tensile _max_σ_y_ stresses had considerably decreased in comparison with the Puddu plate with simple screws, whereas the magnitude of reduction varied between 1.5 and 2 times. The compressive stresses acting in the X- and Z-axes were smaller than those acting in the Y direction by approximately 50% and 30%, respectively, with some exceptions at the lowest and largest wedge sizes (Table 1). For the Puddu plates, the highest tensile stresses were between 2 and 10 times smaller than the compression stresses. The highest magnitudes in the first generation Puddu plate had _max_σ_y_ acting also in the Y-axis at the screw hole just above the osteotomy, and in the Puddu plate with LHS - _max_σ_x_ acting at the conjunction of the tibial fragments (Figure 4). The magnitudes of stresses for most of the points in the four fixation types varied with minor deviations between the studied opening wedge sizes (Table 1). Larger deviations were seen in _min_σ_x,__max_σ_y,_ and _min_σ_y_ for the wedge opening of 22.5 mm. An increase in shear stress with an increase of the wedge size was observable for the Puddu plate with LHS only. For the first generation Puddu plate, the shear stress magnitudes were fluctuating and for both TomoFix plates this stress was initially slightly increasing with increased wedge sizes and then slightly decreasing towards the largest wedge size (Table 1).

There were areas of concentration of tensile and compressive stresses at the screw holes. In the TomoFix plates the largest magnitudes had compressive stresses acting in the plate axis (Z) (Table 1). They were located at the conjunction of the tibial fragments. The compressive and tensile stresses acting in the two other directions (_min_σ_y_, _max_σ_x_) were at least twice as low, and the magnitudes of other stresses were even lower. Two exceptions were tensile stresses (_max_σ_z_) in the TomoFix plate without bone graft at the lowest and the largest opening wedge (Table 1). Where for the Puddu plates, the highest (compressive) stresses were acting at the bone-plate contact point between the upper tibial fragment and the plate insert, for the TomoFix plates the highest (compressive) stresses acted in the conjunction between the tibial fragments (Table 1).

### Displacements

Overall the relative displacement between tibial fragments was low ranging between 0.1 and 24.5 μm for the different fixation options. Comparably, the lowest relative displacement had tibial fragments in the Puddu plate with LHS followed by the first generation Puddu plate, TomoFix plate with bone graft and TomoFix plate without bone graft. With an increasing size of the opening wedge the relative displacement tended to increase for the first three fixation types (Table 2). For the TomoFix plate without bone graft there was a marginal decrease in relative displacement between 24.5 and 22.4 μm when increasing the size of the opening wedge from 7.5 to 17.5 mm. In all fixation options the wedge opening of 22.5 mm was an exception resulting in a (further) decrease of relative displacement.

**Table 2:** The relative displacement of the proximal and distal tibia fragments at different wedge openings

**Fixation**	**Wedge opening (mm)**	**Relative displacement (*μm*)**
First generation Puddu plate	7.5	0.9
	10.5	1.0
	12.5	1.1
	17.5	1.4
	22.5	1.3
Second generation Puddu plate	7.5	0.1
	10.5	0.5
	12.5	0.6
	17.5	1.0
	22.5	0.9
TomoFix plate with bone graft	7.5	7.2
	10.5	8.6
	12.5	9.5
	17.5	10.8
	22.5	8.1
TomoFix plate without bone graft	7.5	24.5
	10.5	24.0
	12.5	23.5
	17.5	22.4
	22.5	17.1

### Stresses in plates

Table 3 shows stresses occurring in the plates. The highest stresses were either compressive stresses acting in the plate axis (Z) or shear stresses (von Mises stress). For the Puddu plate with LHS and for the TomoFix plate with bone graft the compression stresses were mostly higher than the shear stresses. For the Puddu plate with simple screws and for the TomoFix plate without bone graft the shear stresses were higher than the compressive ones.

**Table 3:** The stresses in the plates at different wedge openings

**Fixation**	**Wedge opening (mm)**	_**max**_**σ**_**z **_**(tensile stress)**	_**min**_**σ**_**z **_**(compression stress)**	_**mis**_**σ (share von Mises stress)**
First generation Puddu plate	7.5	10.2	−31.9	37.5
	10.5	15.8	−38.1	48.6
	12.5	18.9	−33.2	42.4
	17.5	22.9	−34.9	49.7
	22.5	34.3	−28.9	34.7
Second generation Puddu plate	7.5	46.2	−55.3	43.2
	10.5	45.9	−52.5	42.9
	12.5	45.5	−48.5	47.2
	17.5	44.1	−43.4	48.2
	22.5	55.9	−69.0	52.6
TomoFix plate with bone graft	7.5	35.0	−94.1	91.8
	10.5	37.5	−99.1	97.7
	12.5	39.0	−104.0	102.0
	17.5	41.5	−109.0	108.0
	22.5	42.0	−115.0	110.0
TomoFix plate without bone graft	7.5	79.3	−200.0	205.0
	10.5	80.6	−197.0	204.0
	12.5	79.4	−197.0	205.0
	17.5	79.5	−193.0	205.0
	22.5	79.9	−200.0	208.0

The compressive stresses were higher as the tensile stresses in the plates with probably one notable exception of the largest wedge opening for the first generation Puddu plate, in which the magnitude of the tensile stress was about 15% higher than that of the compressive stress.

**Figure 5:**
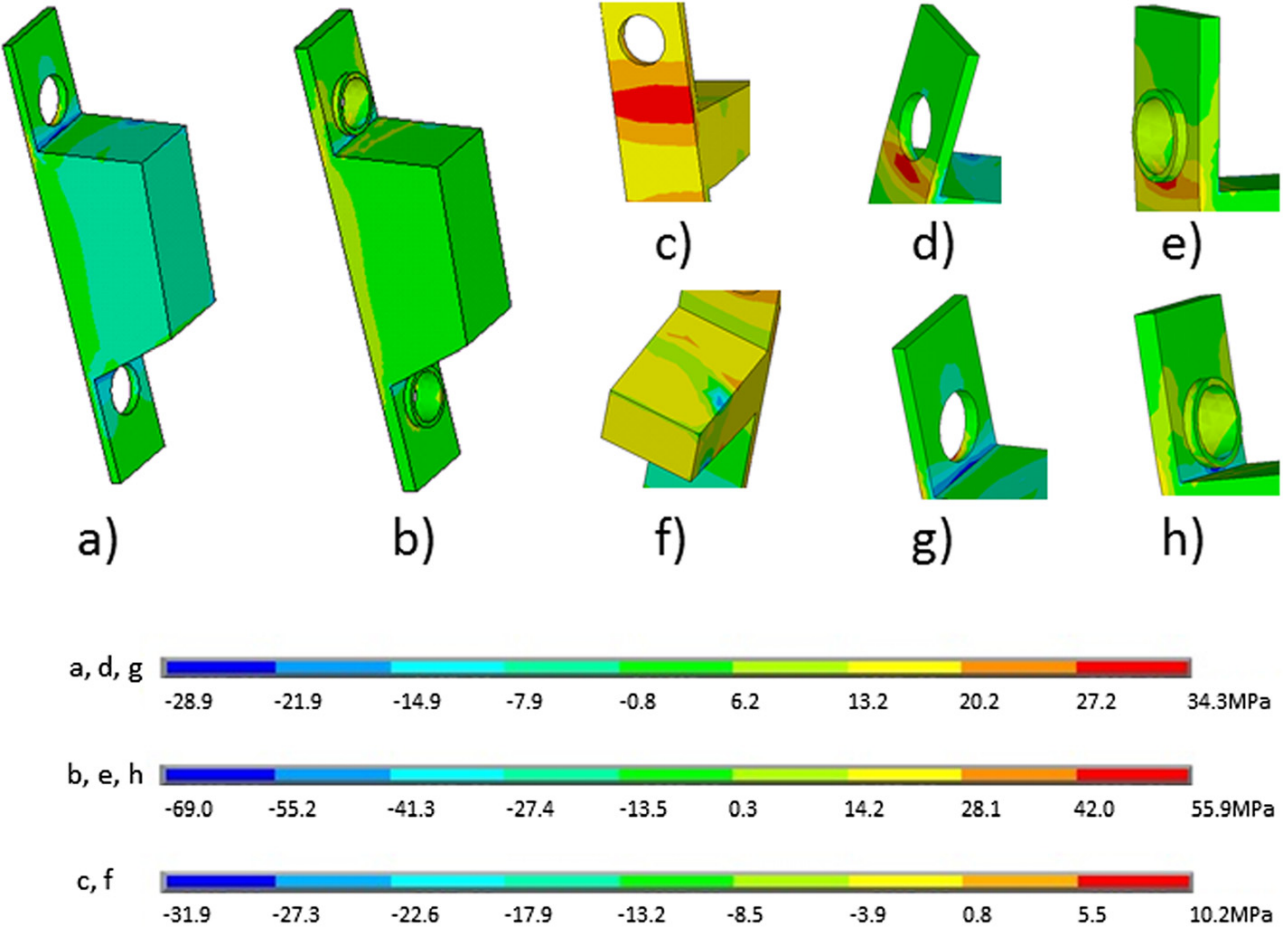
**The distributions of σ**_**z **_**stresses in the (a) first and (b) second generation Puddu plates in the entire volume and in zones of stress concentration (c-h).**

For the first generation Puddu plate the magnitudes of compressive and shear stresses were fluctuating with increasing wedge size, but the tensile stresses were clearly increasing. For the Puddu plate with LHS with an increasing wedge size the shear stresses tended to increase, compression stresses tended to decrease and tensile stresses tended to marginally decrease with one exception for the largest wedge size, in which the compressive and the tensile stress were the highest, respectively (Table 3). For the TomoFix plate with bone graft all stresses were increasing with increasing wedge size. Finally, the TomoFix plate without bone graft stresses did not seem to depend on wedge size.The distribution of stresses was clearly non-uniform (Figure 5). For the first generation Puddu plate, the zones of concentration of compressive stresses were located at the contact points between the metal insert and cortical bone on the side that is opposite to the plane of symmetry (Figure 5f). The zone of concentration of tensile stress was located on the outer side of the plate, in the opposite of the upper bound of the metal insert (Figure 5c). The location of points of stress concentration was valid for all wedge openings except for the largest wedge opening in which these points changed: the point of maximum tensile stress was located closer to the edge of the screw holes (Figure 5d), and the maximum compressive stresses occurred at the boundary between the plate and the metal insert (Figure 5g).

**Figure 6:**
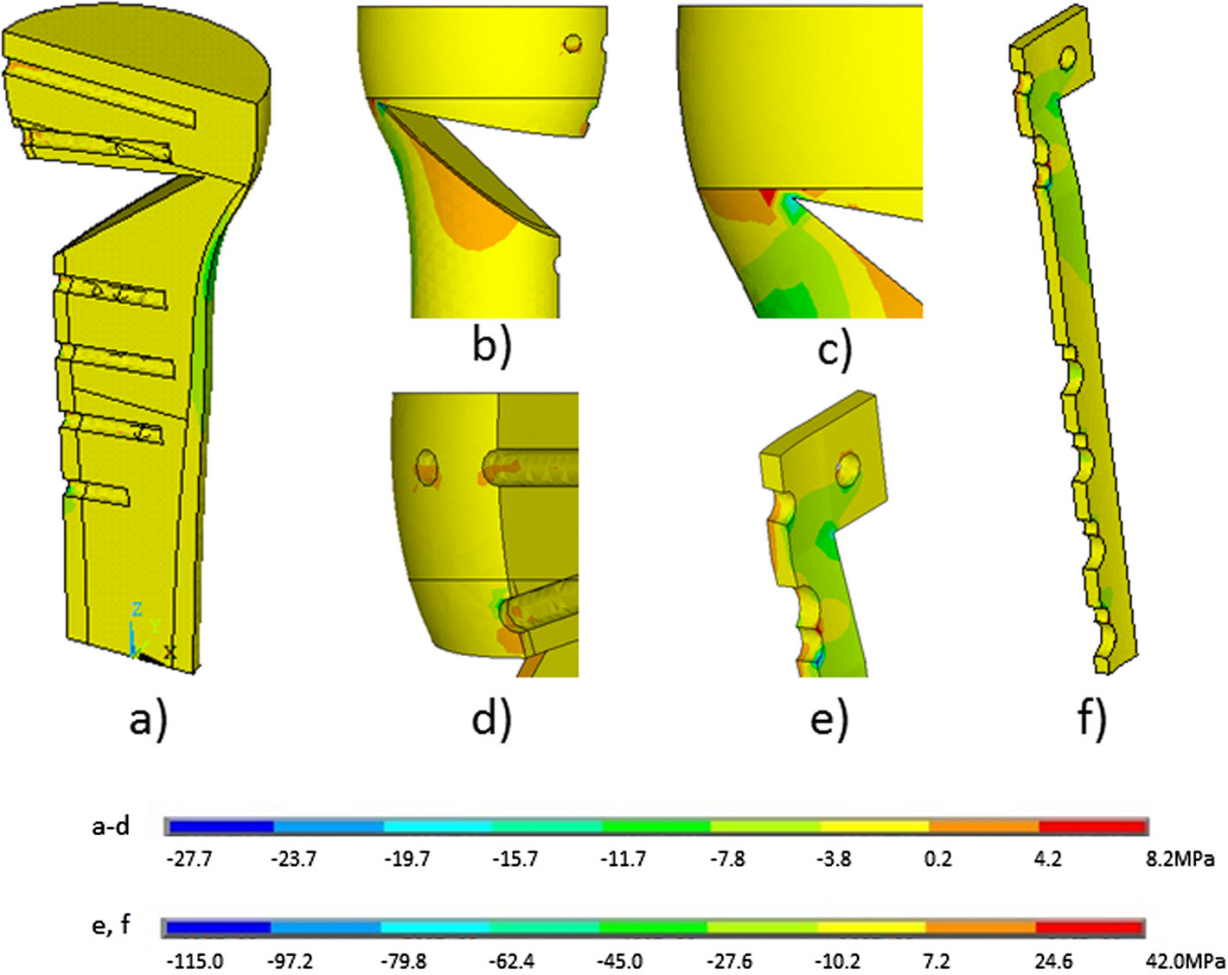
**The distribution of stresses σ**_**z **_**in the bone (a-d) and the TomoFix plate (e, f) with bone graft.**

For the Puddu plate with LHS, the increase in the magnitudes of the stresses was associated with the narrowing of the zones of stress concentration. In the first generation Puddu plate the stress concentration zones were less precisely defined, and in the Puddu plate with LHS, those zones were narrowed to the boundary between the cylindrical insert and the surface of the plate. The maximum compressive stress occurred on the inner side (Figure 5h) and the maximum tensile stress on the outer side (Figure 5e) of the plate. In the TomoFix plate with bone graft, those maximum stresses appeared at the screw hole just above the osteotomy on the inner side of the plate (Figure 6). A comparison of the TomoFix plate fixations with and without bone graft showed that in the latter case all stresses had almost doubled (Table 3), but the stress distribution in the plate did not change.

### Screws

It can be seen in Table 4 that in all fixation types the occurring tension pulled the screws and exceeded the allowable values of 1.2 MPa at least on one of the studied screw surfaces. While the screws in the first generation Puddu plate exceeded the allowable values of 1.2 MPa by factor 3.8, the screws in the Puddu plate with LHS exceeded the threshold by factor 5, the screws in the TomoFix plate with bone graft by factor 2.2, and the screws in the TomoFix plate without bone graft by factor 6. The stress excess for the screws of the TomoFix plate with bone graft occurred at one out of twelve surfaces of the six studied screw channels (Table 4). For the TomoFix plate without bone graft a stress excess occurred at five out of twelve surfaces of the six screw channels. For the first generation Puddu plate a stress excess occurred at all four screw surfaces of the two screws.

**Table 4:** Stresses along the screw channels in bone

**Fixation**	**Screw channel (from proximal)**	**Surface of the channel**	_**max**_**σ**_**x **_**(compression stress)**	_**min**_**σ**_**x **_**(tensile stress)**
First generation Puddu plate	1	Upper	**2.16**	
		Lower		**2.07**
	2	Upper		**2.57**
		Lower		**4.6**
Second generation Puddu plate	1	Upper	1.50	
		Lower		**6.04**
	2	Upper		**1.47**
		Lower		**5.23**
TomoFix with bone graft	1	Upper		0.34
		Lower		0.41
	2	Upper	0.78	
		Lower		**2.58**
	3	Upper		0.28
		Lower		1.20
	4	Upper		0.55
		Lower		1.08
	5	Upper		0.34
		Lower		0.87
	6	Upper		0.25
		Lower		0.48
TomoFix without bone graft	1	Upper		0.53
		Lower		0.58
	2	Upper	0.78	
		Lower		**7.15**
	3	Upper		**2.20**
		Lower		**4.70**
	4	Upper		**1.24**
		Lower		**2.10**
	5	Upper		0.46
		Lower		0.98
	6	Upper		0.35
		Lower		0.76

Note: the values exceeding the maximum allowable threshold for the stresses on the screw are in bold.

## Discussion

The main findings of this study were that for the vast majority of the opening wedge sizes and fixation types the shear stress was dominating in the bone independent of fixation type. With an increasing size of the wedge the relative displacement of tibial fragments tended to increase in all fixation types except in the TomoFix without bone graft, in which the relative displacement did marginally decrease. This was despite the fact that the thickness of the cortical layer was modelled as increasing towards distally, which means that larger wedge osteotomies should have had even stronger cortical layer for the fixation of the distal tibial fragment. The increase of relative displacement with an increasing wedge size can be physically anticipated. When analysing the model of the TomoFix plate without bone graft in more detail, it became obvious that the displacement of the proximal tibial fragment in relation to the plate remained almost unchanged and the displacement of the distal tibial fragment in relation to the plate increased. This might potentially explain the marginal decrease of the relative displacement of tibial fragments to each other with an increasing wedge size. Large opening wedge sizes such as 22.5 mm are rather rare in clinical practice, considering the average width of the tibia. When observing the distribution of stresses in bone and in plates for both Puddu plates this large wedge size tended to break ranks. This may potentially be associated with the change in the point of maximum stress and may mean rather unfavourable fixation conditions for the plates due to increased stresses. In a failure analysis of a consecutive case series by Nelissen et al. a wedge size over 10 mm showed a significantly higher overall complication rate [22].

The highest stresses in the plates were either compressive ones acting in the plate axis or shear stresses perpendicular to the plate. The magnitudes of stresses observed in the TomoFix plates were higher than those seen in the Puddu plates. This observation was also reported in the FE analysis by Raja Izaham et al. [13]. The observed increase of compressive stresses can be attributed to the design of the plate, namely to the connection between the screws and the plate. Those stresses directed in the plate axis represent the major risk for potential hardware failure or instability. This risk is, however, less pronounced for the TomoFix plate, since it is fixed in the proximal tibial fragment using four screws. Similarly, constructs without locking head screws resist the stresses in the sagittal plane to a lesser extent, even in the presence of a metal insert.

An excess of the allowable stress on the screw surfaces occurred in each of the four studied fixation types. The lowest ratio (1 out of 12 surfaces) was observed for the TomoFix with bone graft, followed by the TomoFix without bone graft (5/12) and the Puddu plate with LHS (3/4). An excess of the allowable stress on all surfaces (4/4) was seen in the first generation Puddu plate.

The overall stresses in the bone were lower for the TomoFix plate with bone graft and the Puddu plate with LHS, followed by the TomoFix plate without bone graft and the first generation Puddu plate. This shows a higher fixation stability of the first three constructs and a lower comparative fixation stability of the first generation Puddu plate. Compared with the first generation Puddu plate with imperfect joints connection, the model of the second generation was 0.5 mm thicker, had locking head screws with cylindrical insert and a hard screw-plate connection. This should have contributed to the higher stability of the second generation Puddu plate. Similarly, the modelled bone graft with the averaged parameters of the cortical and cancellous bone in the TomoFix plate has contributed to additional fixation stability of the TomoFix model with bone graft. However, although the model appears to be rather recommendable based on the results of the FE analysis, one should bear in mind donor site morbidity, potential infections or other complications when using a bone graft [23,24]. Brinkman et al. proposed to use autologous bone but only for filling gaps >20 mm when a stable fixation can be warranted [7]. Staubli et al. and Brosset et al. achieved good clinical results in their case series with the TomoFix plate and no grafts [3,15]. Also other clinical [25] and biomechanical [8] studies emphasized good fixation stability of the TomoFix plate. In our model, the modelled bone graft added mechanical stability; however, in clinical practice the role of a bone graft may be less important for adding mechanical stability but rather for accelerating bone healing. No prospective randomized trials have yet been published that compare the various filling materials with no filling at all.

The FE analysis by Raja Izaham et al. compared Puddu and TomoFix plates with a 10 mm opening wedge [13]. The authors concluded that the rigidity and therefore stability of the TomoFix system was better than that of the Puddu plate system [13]. Their modelling methods were different, however, from those in our study. The FE analysis by Luo et al. calculated stresses in the TomoFix plate around 171 MPa, which were comparable to those in our study (e.g. around 100 MPa for the TomoFix plate with bone graft and around 200 MPa for the TomoFix plate without bone graft) [14]. The authors suggested that a one-leg plate system with locking screws can be used for the majority of patients without heavy bodyweight and poor bone quality.

The first generation Puddu plate seemed to have the comparably lowest fixation stability. Although biomechanical tests confirmed the adequacy of the fixation and load stability of the Puddu like plates with an insert and without locking head screws [9-11], some authors raised concerns with the first generation Puddu plate in clinical case series [22,26,27]. On the other hand, the biomechanical study by Stoffel et al. concluded that the TomoFix plate provides superior stability in both compression and torsion compared to the Puddu Plate and that in the latter case additional fixation might be required [12].

The stresses in the plates were much higher than those in the bone but the material properties of plates are also more robust compared with bone. The maximum stress up to 208 MPa was observed in the TomoFix plates. The yielding strength of stainless steel, which is the material of the Puddu plates, is 750 MPa [14], and that of the titan alloy used in the TomoFix plate is even higher. Still, hardware failure is not infrequently reported in the literature. Spahn et al. reviewed the results of 85 MOWHTOs, which were stabilized with either a first-generation Puddu plate (n = 55) or a C-plate (n = 30) [27]. The authors observed nine hardware failures in the Puddu plate group (two plate breakages, seven screw breakages) compared with none for the C-plate [27]. Valkering et al. studied complications after MOWHTO using TomoFix plates in 40 patients [25]. Only one hardware failure, which was a screw fracture during implant removal, occurred [25]. These results are in accordance with the calculated stresses in the respective plates and screws in our study.

One of the limitations of the MOWHTO is a relatively long rehabilitation period with crutches for around 6–8 weeks. A loss of fixation stability during these two months may compromise healing. A more rigid fixation type leading to an earlier weight bearing is clearly desired. Brinkman et al. showed in his consecutive series of 14 osteotomies with angle-stable TomoFix fixation that early full weight bearing, as soon as pain and wound healing permits (probably two weeks postoperative), has good clinical outcome without the feared loss of correction [28]. Similarly, Takeuchi et al. were able to show good clinical results in 57 osteotomies with TomoFix plate and an early full weight bearing exercise program two weeks postoperative [29]. The authors did not observe loss of correction, but documented a tibia plateau fracture in one case and a lateral cortical fracture in another.

### Limitations

The plates were compared and studied under full weight bearing conditions. Although physically active subjects may develop maximal compression forces in the knee joint that exceed full weight bearing conditions up to factor seven [30], from the clinical point of view full weight bearing is not prescribed at least in the early rehabilitation stages. Furthermore, 842 N were assumed as screw extraction strength based on the results of Hearn et al. [21]. The screw extraction strength required for screw failure may depend on the biomechanical test settings. To avoid a non-essential complexity of the bone model, a cortical layer with constant properties between the levels h1 and h4 and no cortical layer between the h4 and h5 level was assumed. Moreover, for the purpose of rational use of computer resources a geometric plane of symmetry of the plate was studied. Similarly, while assessing the stresses in the screw channels in the TomoFix plates, only six screw holes (out of the eight TomoFix screw holes in total) lying in the axis Z were considered to assess a symmetrical problem.

## Conclusions

Different fixation devices generate different fixation stability profiles for various opening wedge sizes. Based on the computational simulations, none of the studied osteosynthesis fixation types warranted an intransigent full weight bearing per se. The stresses along the screw channels exceeded the allowable limit in all devices. Since the highest load in all fixation types acted in the sagittal plane, two or more screws are required for fixation of the proximal tibial fragment. The highest fixation stability was observed for the TomoFix plates and the lowest stability for the first generation Puddu plate without locking head screws. Based on the results of the current study, the TomoFix plate seems the implant of choice for a rigid fixation. These findings were revealed in theoretical models and need to be validated in controlled clinical setting.

## Abbreviations

CT: Computer tomography; FE: Finite element; MOWHTO: Medial opening wedge high tibial osteotomy; LHS: Locking head screws.

## Competing interests

The authors declare that they have no competing interests.

## Authors’ contributions

MLG conceived the study, interpreted the results and drafted the manuscript. WO and KPB participated in the design of the study and interpretation of the results. SP performed the finite element analysis. PB and PH assisted in interpreting the clinical implication and biomechanical results of the study. EA participated in the design of the study, interpreted the study results, performed the literature search and supervised the drafting of the manuscript. All authors read and approved the final manuscript.

## Pre-publication history

The pre-publication history for this paper can be accessed here:

http://www.biomedcentral.com/1471-2474/15/230/prepub
